# Global reductions in seafloor biomass in response to climate change

**DOI:** 10.1111/gcb.12480

**Published:** 2013-12-31

**Authors:** Daniel O B Jones, Andrew Yool, Chih-Lin Wei, Stephanie A Henson, Henry A Ruhl, Reg A Watson, Marion Gehlen

**Affiliations:** 1 National Oceanography Centre, University of Southampton Waterfront CampusEuropean Way, Southampton, SO14 3ZH, UK; 2 Ocean Science Centre, Memorial University of NewfoundlandSt. John's, A1C 5S7, NL, Canada; 3 Institute for Marine and Antarctic Studies Taroona, University of TasmaniaPrivate Bag 49, Hobart, Tasmania, 7001, Australia; 4 LSCE/IPSL, Laboratoire des Sciences du Climat et de l'Environnement, CEA-CNRS-UVSQ Orme des MerisiersBât. 712, CEA/Saclay 91198, Gif-sur-Yvette, Cedex, France

**Keywords:** benthic, deep-sea, macroecology, macrofauna, megafauna, meiofaunal, size structure, standing stock

## Abstract

Seafloor organisms are vital for healthy marine ecosystems, contributing to elemental cycling, benthic remineralization, and ultimately sequestration of carbon. Deep-sea life is primarily reliant on the export flux of particulate organic carbon from the surface ocean for food, but most ocean biogeochemistry models predict global decreases in export flux resulting from 21st century anthropogenically induced warming. Here we show that decadal-to-century scale changes in carbon export associated with climate change lead to an estimated 5.2% decrease in future (2091–2100) global open ocean benthic biomass under RCP8.5 (reduction of 5.2 Mt C) compared with contemporary conditions (2006–2015). Our projections use multi-model mean export flux estimates from eight fully coupled earth system models, which contributed to the Coupled Model Intercomparison Project Phase 5, that have been forced by high and low representative concentration pathways (RCP8.5 and 4.5, respectively). These export flux estimates are used in conjunction with published empirical relationships to predict changes in benthic biomass. The polar oceans and some upwelling areas may experience increases in benthic biomass, but most other regions show decreases, with up to 38% reductions in parts of the northeast Atlantic. Our analysis projects a future ocean with smaller sized infaunal benthos, potentially reducing energy transfer rates though benthic multicellular food webs. More than 80% of potential deep-water biodiversity hotspots known around the world, including canyons, seamounts, and cold-water coral reefs, are projected to experience negative changes in biomass. These major reductions in biomass may lead to widespread change in benthic ecosystems and the functions and services they provide.

## Introduction

The ocean represents the largest habitat on earth, covering around 71% of the Earth's surface (Watling *et al.*, 2013), and the global seafloor harbours a living biomass of around 110 MtC, of which over half is in water deeper than 3000 m (Wei *et al.*, 2010). Upper ocean biomass is projected to decrease in response to surface ocean warming (Joos *et al.*, 1999; Steinacher *et al.*, 2010), as increased stratification and slowed mixing reduces the nutrient supply for primary production at the ocean's surface. In turn, this is expected to reduce surface ocean production (Bopp *et al.*, 2001; Steinacher *et al.*, 2010) and hence the flux of particulate organic carbon (POC) from the surface to benthic communities. As this flux provides the major food input to open ocean seafloor communities (McClain *et al.*, 2012) and because most deep-water communities are limited by food supply (Young & Eckelbarger, 1994; Smith *et al.*, 2008a), 21st century climate change and variation in food supply are likely to drastically alter benthic community biomass, composition and functioning (Billett *et al.*, 2001; Ruhl & Smith, 2004; Smith *et al.*, 2008b, 2013).

Major change in the open ocean benthos is likely to have profound direct and indirect impacts on biologically controlled processes, ecosystem functioning, and the services these systems provide. Changes in POC flux can impact the diversity of organisms within a habitat (Ruhl & Smith, 2004), and projected future reductions will likely result in changes in the taxonomic composition of deep-water fauna as well as the species richness and distribution of biodiversity in the world's oceans (Levin *et al.*, 2001). The deep ocean contains many key habitats and ecosystems of conservation priority, which are often hot spots for biodiversity and biomass, including cold-water coral reefs, seamounts, canyons, and ridges (Ramirez-Llodra *et al.*, 2010). The deep-sea provides direct services and benefits for humans, for example, deep-sea fisheries are an important, although declining, resource (Devine *et al.*, 2006) and deep-sea species likely harbour stocks of new biologically active compounds for pharmaceutical development (Skropeta, 2008). Although exchanges between the deep ocean and surface waters are typically slow, the benthic fauna play a role in remineralization, bioturbation, sediment mixed layer depth, sediment community oxygen consumption, and ultimately carbon burial (Smith *et al.*, 2008a,b). Thus, on centennial to millennial timescales, major changes in biomass are likely to influence nutrient regeneration and calcite saturation levels in the ocean (Archer & Maier-Reimer, 1994; Smith *et al.*, 2008a).

Here we show how projected century scale climate change affects global oceanic primary production, export of POC to the seafloor, and ultimately changes in benthic biomass. Output from eight fully coupled earth system models (ESM), which contributed to the 5th Coupled Model Intercomparison Project (CMIP5), was evaluated and used to construct a multi-model mean of export flux. Output from projections from the same models forced by two different Representative Concentration Pathways (RCP4.5 as moderate and RCP8.5 as a high radiative forcing scenario) was then used to characterize the change in export flux during the 21st century. Benthic biomass under these scenarios was projected using empirical relationships between POC flux to the seafloor and the biomasses of three metazoan size classes: meiofauna, macrofauna, and megafauna (Wei *et al.*, 2010). The impacts of projected changes to key deep-water habitats, such as seamounts and canyons, and important deep-water fishing areas are assessed.

## Materials and methods

Export production (EP) fields from eight earth system models (ESMs) and a multi-model mean were used for analysis. EP was converted to POC flux to 500 m above the seafloor using the Martin curve (Martin *et al.*, 1987; Amante & Eakins, 2009). Empirical relationships between POC flux at 500 m above the seafloor and biomass of three benthic faunal size classes (Wei *et al.*, 2010) (meiofaunal, macrofauna, and megafauna), developed with reference to a major dataset on benthic fauna collected by the Census of Marine Life, were applied to the model output.

### Earth system models

The models used in this study (Table 1) were fully coupled, three-dimensional atmosphere ocean climate models that included representations of the marine and terrestrial carbon cycle (Friedlingstein *et al.*, 2006). The models selected contributed to the IPCC AR5 exercise. For the recent exercise, models were forced by atmospheric CO_2_ concentrations rather than emissions. The time course of atmospheric CO_2_ was reconstructed from observations over the historical period up to the year 2005 and follows Representative Concentration Pathways (RCP) until 2100 (Meinshausen *et al.*, 2011). RCPs describe a possible range of radiative forcing values (e.g., RCP8.5 = 8.5 W m^−2^) in the year 2100. Here two RCPs are used: RCP4.5 (medium-low) and RCP8.5 (high), which yield an atmospheric CO_2_ concentration in 2100 of 538 and 936 ppm respectively (Meinshausen *et al.*, 2011). The latest carbon dioxide emissions suggest that RCP8.5 is the most realistic scenario (Peters *et al.*, 2013); therefore, we focus on the results using RCP8.5. The data from RCP4.5 are available in the Supporting information for comparison.

**Table 1:** Details of the earth system models used in analysis. Model details include the abbreviated name used through the manuscript and the full name, institute, and reference. Global integrated export (EP100) values are provided for each model for the period 2006–2015 under moderate (RCP4.5) and severe (RCP8.5) scenarios

Model	Full name	Institute	Reference	Present total global export, Gt C yr^−1^	
				RCP4.5	RCP8.5
IPSL CM5-MR	Climate Model 5 – Medium Range	Institut Pierre Simon Laplace	Dufresne *et al*. (2013)	7.09	7.15
IPSL-CM5-LR	Climate Model 5 – Long Range	Institut Pierre Simon Laplace	Dufresne *et al*. (2013)	6.68	6.73
MPI-ESM-LR	Earth System Model – Long Range	Max-Planck Institute for Meteorology	Giorgetta *et al*. (2013)	7.94	7.89
CESM	Community Earth System Model, version 1–Biogeochemistry	National Center for Atmospheric Research	Moore *et al*. (2013)	7.65	7.69
CNRM-CM5	Climate Model 5	Centre National de Recherches Météorologiques	Voldoire *et al*. (2013)	4.43	4.47
CanESM2	Canadian Earth System Model	Canadian Centre for Climate Modelling and Analysis	Chylek *et al*. (2011)	10.73	10.63
GFSL-ESM2M	Earth System Model – Modular Ocean Model version	National Ocean and Atmospheric Administration Geophysical Fluid Dynamics Laboratory	Dunne *et al*. (2012)	7.07	7.08
HadGEM2-CC	Global Environment Model 2 – Carbon Cycle	UK Met Office Hadley Centre	Collins *et al*. (2011)	5.48	5.48

Export production estimates at 100 m depth (EP100) from these eight ESMs and a simple multi-model mean were used for analysis. Mean monthly export values were assessed for two time periods, 2006–2015 and 2091–2100.

### Data standardization

Gridded bathymetry data (ETOPO1) were obtained from the National Oceanic and Atmospheric Administration National Geophysical Data Center (Amante & Eakins, 2009). Data were reduced to 1° resolution by averaging (mean) cells in the original 1 arc-minute grid.

Data from the ESMs were supplied on grids of different resolution and geographic projection. For ease of handling, all data were regridded by averaging (mean) to a standard 1°grid (latitude −90 : 90°N; longitude −180 : 180°E).

### Model validation

To validate the various ESMs, modelled EP100 was compared with satellite-derived estimates of export production. As *in situ* observations of EP100 are relatively sparse in both space and time, satellite-based estimates of primary production (NPP) and SST were converted to export (EP100obs) using three algorithms (Laws *et al.*, 2000; Dunne *et al.*, 2007; Henson *et al.*, 2012). Observations, and model output, were averaged from the period 1998 to 2007, the first continuous decade of synoptic satellite coverage of ocean colour (from which primary production is estimated). The models were compared with these observed data using Taylor (2001) diagrams.

### Data processing

Flux to the seafloor was calculated using the Martin curve (Martin *et al.*, 1987) with a *b* value of −0.858, applied to modelled export production (EP100) and depth (ETOPO1) as inputs (export depth was 100 m).


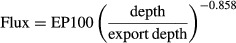
 Flux to 500 m above the seafloor was also calculated (see later section) in a similar manner. As such, all grid cells with water depth shallower than 500 m were excluded from analysis.

Benthic biomass was inferred from flux at 500 m above the seabed [poc.flx.mean = POC flux at 500 m above seafloor (mg C m^−2^ day^−1^)] using a previously published highly significant statistical relationship (*P* < 0.001) between a large spatially referenced database of benthic biomasses (mg C m ^−2^) of meiofauna (mei.biom), macrofauna (mac.biom), and megafauna (meg.biom) and POC flux at 500 m above seafloor estimated from satellite data (Wei *et al.*, 2010). A relationship between bacteria (bac.biom) and POC flux at 500 m above seafloor was determined, but was not significant (*R*^2^ < 0.001, *P *= 0.25), so was not used. The relationships used were:

log 10 (mei.biom) = 1.4347 + 0.4428 log 10(poc.flx.mean).
log 10 (mac.biom) = 1.8422 + 0.6655 log 10(poc.flx.mean).
log 10 (meg.biom) = 1.4687 + 0.3948 log 10(poc.flx.mean).

Total biomass values (mg C m^−2^) were obtained by summing the biomasses for meiofauna, macrofauna, and megafauna. Global total biomasses were calculated separately for metazoans (excluding bacteria) and for all fauna (including bacteria). All graphical figures are presented without bacteria. Where bacteria were included in totals this was done by adding a fixed benthic biomass of bacteria dataset, calculated from the Census of Marine Life data (Wei *et al.*, 2010). There is currently insufficient evidence to determine climate-related changes in bacterial biomass, because the bacterial populations are relatively invariant globally and across different flux conditions (Wei *et al.*, 2010), potentially reaching maximum possible biomass in the porous sedimentary matrix (Schmidt *et al.*, 1998). As the bacterial biomass value was assumed to be constant across the time series, absolute change figures were identical with or without the addition of the bacterial biomass. Percentage changes were reduced with the addition of the bacterial biomass.

Global biomasses, or biomasses within depth bands, are expressed in units of mg C and were calculated by summing the within grid-cell total biomasses. Within cell total biomasses were calculated by multiplying cell biomasses (in units of mg C m^−2^) by the area of each cell (m^2^).

Percentage differences between average present (2006–2015) and average future (2091–2100) fluxes were used to assess regions that would experience the greatest potential change in benthic biomass.

Two estimates of error were made. One assessed the error in the biomass flux relationships used and the other assessed variation between the models. The error in the biomass flux relationships was calculated as the standard error of the regression for each size class independently. Predictions were made for each projected flux value following standard methods for estimating standard error for *y* for a given value of *x* from a linear regression (Sokal & Rohlf, 1995). The coefficient of variation between multiple model estimates was calculated as the ratio of the standard deviation of the eight models and the mean over a 10-year period (either 2006–2015 or 2091–2100). The errors for biomass of individual size classes were summed to give total errors.

The global ocean basin extents of the Atlantic, Pacific, and Indian oceans (without their Arctic or Southern Ocean extensions) were defined using the World Ocean Atlas (from the NOAA National Oceanographic Data Center) basins. The Arctic Ocean was defined as the area north of 66°N (the geographical Arctic). The Southern Ocean was taken as the area south of 60°S to coincide with the approximate position of the Antarctic convergence (International Hydrographic Organization, 2002).

The areas of the world with features of interest, such as cold-water coral reefs, were extracted from global datasets to make an initial quantification of the projected impacts to these important areas. For each area of interest, a binary mask was made at 1° resolution indicating presence or absence of the feature of interest. The areas with seamounts were assessed from a high-resolution vector (polygon) database of seamount base areas (Yesson *et al.*, 2011). The 1° seamount presence data were inclusive; a 1° cell was defined as having a seamount if any part of the base of any seamount was present within the cell. A similar approach was taken with canyons and cold-water coral reefs. The datasets used in these cases were a vector database of canyon centre lines (Harris & Whiteway, 2011) and a point database of cold-water coral reef occurrence (Freiwald *et al.*, 2005). To calculate the areas of importance to deep-sea fisheries, a 1° gridded database of average annual catch rates of deep-sea fishes (tonnes km^−2^ yr^−1^) was used (Watson *et al.*, 2004) that had been updated for the year 2006 (the most recent available). The area assumed to have fishing importance was defined here as the 1° cells where fishing occurs at a rate of >1 t km^−2^ yr^−1^.

## Results

**Figure 1:**
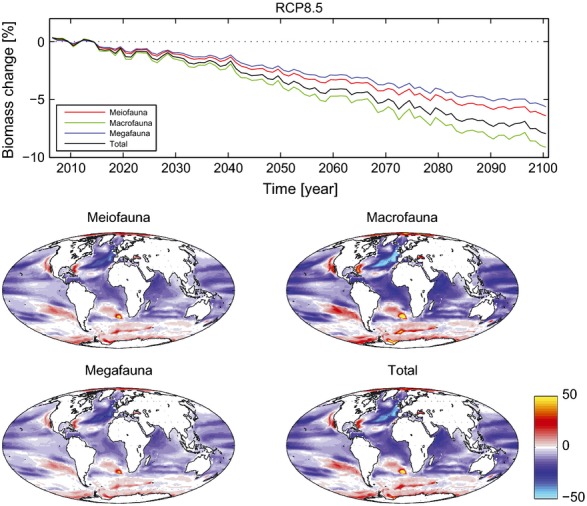
Changes in benthic biomass between 2006–2015 and 2091–2100 under scenario RCP8.5. Panel 1 shows projected changes in biomass of metazoan size-categories of benthos across the modelled time series (as annual means). Panels 2–5 show maps of percentage changes in multi-model mean benthic biomass on seafloor (mg C m^−2^). Benthic biomasses presented as totals (metazoans only) and split into three size classes. The map projection is Mollweide equal area projection.

**Figure 2:**
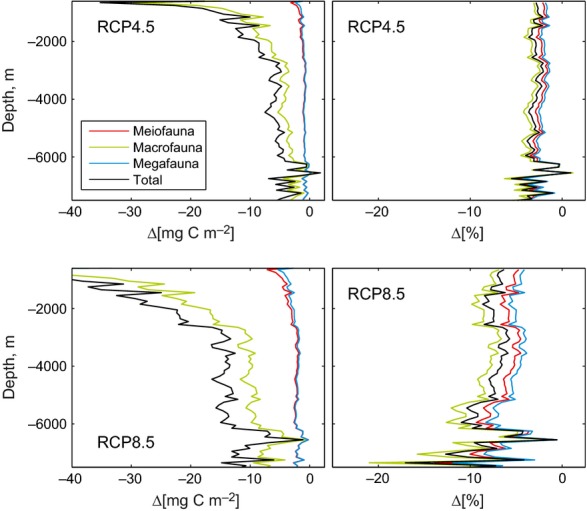
Patterns relating to seafloor depth in multi-model mean biomass change between 2006–2015 and 2091–2100. Biomass presented as total (metazoan only) and split into three size classes. Left panel shows absolute change in biomass (Δ; mg C m^−2^) and the right panel shows percentage change. Projected relationships shown under moderate scenario (RCP4.5; top) and severe scenario (RCP8.5; bottom).

Global export of POC from surface waters decreases over time in all projections. These changes in export lead to substantial reductions in predicted POC flux to the seafloor. With a reduced supply of organic material, our projections suggest that total global seafloor biomass will decrease by 5.2%, as the biomass of all size classes of benthic metazoan fauna decreases in this century (Fig.1; Table 2). The projections show a general shift in biomass to smaller size classes of benthic infauna, particularly in abyssal waters (Fig.2). Macrofauna (here defined as 250–520 μm in size, e.g., polychaetes) are the dominant metazoans in terms of biomass. Macrofaunal biomass decreases in projections by a greater percentage than meiofaunal (20–250 μm size, e.g., nematodes) and megafaunal (epibenthic invertebrates, e.g., echinoderms and demersal fishes >10 mm) biomass. Although total biomasses of megafauna and meiofaunal are similar, projected megafaunal biomass reductions are greater than for meiofauna.

**Table 2:** Changes in POC flux and biomass between 2006–2015 and 2091–2100 under scenario RCP8.5. Changes in specific regions and depth bands for POC flux to seafloor (Mt C yr^−1^) and biomass (MtC). Values presented are absolute change between present and future projections. The greatest positive and negative changes are local values (per 1° cell) in units of mol C m^−2^ yr^−1^ for POC flux and g C m^−2^ for biomass. Percentage changes are presented in parentheses. For local areas of greatest positive and negative change regional location codes are given: NA, North Atlantic; Ar, Arctic

Area	POC flux to seafloor	Meiofauna	Macrofauna	Megafauna	Metazoan Total	Total (with bacteria constant)
Globe	−58.263 (−11.4%)	−0.773 (−5.87%)	−3.771 (−8.38%)	−0.694 (−5.15%)	−5.238 (−7.31%)	−5.238 (−5.21%)
Atlantic	−25.937 (−15.4%)	−0.258 (−7.85%)	−1.230 (−11.2%)	−0.230 (−6.86%)	−1.788 (−9.83%)	−1.788 (−7.23%)
Pacific	−44.519 (−5.51%)	−0.754 (−3.01%)	−3.605 (−4.27%)	−0.682 (−2.64%)	−5.041 (−3.73%)	−5.041 (−2.63%)
Indian	−26.937 (−3.98%)	−0.516 (−2.20%)	−2.446 (−3.07%)	−0.466 (−1.94%)	−3.428 (−2.70%)	−3.428 (−1.87%)
Arctic	−4.059 (−12.6%)	0.001 (0.41%)	−0.024 (−2.67%)	0.002 (0.94%)	−0.020 (−1.46%)	−0.020 (−1.18%)
Southern	2.936 (9.64%)	0.033 (4.41%)	0.167 (6.50%)	0.029 (3.84%)	0.229 (5.62%)	0.229 (4.02%)
Bathyal	−15.607 (−10.9%)	−0.091 (−5.58%)	−0.620 (−8.00%)	−0.074 (−4.87%)	−0.785 (−7.20%)	−0.785 (−5.75%)
Abyssal	−31.433 (−11.9%)	−0.676 (−5.91%)	−3.128 (−8.46%)	−0.614 (−5.17%)	−4.418 (−7.33%)	−4.418 (−5.13%)
Hadal	−0.172 (−11.9%)	−0.006 (−7.20%)	−0.023 (−9.57%)	−0.006 (−6.42%)	−0.035 (−8.39%)	−0.035 (−5.52%)
Area with fishing	−23.515 (−14.5%)	−0.088 (−6.72%)	−0.546 (−9.57%)	−0.074 (−5.90%)	−0.708 (−8.56%)	−0.708 (−6.44%)
Area with seamounts	−16.317 (−13.8%)	−0.354 (−7.02%)	−1.639 (−9.95%)	−0.322 (−6.15%)	−2.314 (−8.66%)	−2.314 (−6.06%)
Area with canyons	−9.512 (−11.2%)	−0.105 (−6.11%)	−0.616 (−8.64%)	−0.090 (−5.36%)	−0.810 (−7.71%)	−0.810 (−5.64%)
Area with cold- water corals	−5.345 (−20.9%)	−0.028 (−9.14%)	−0.152 (−12.5%)	−0.024 (−8.09%)	−0.204 (−11.2%)	−0.204 (−8.64%)
Greatest positive change	79 498.3 (62.32%) NA	88.432 (22.61%) NA	822.882 (36.79%) NA	65.935 (19.22%) NA	977.249 (28.65%) NA	977.249 (25.84%) Ar
Greatest negative change	−367 904 (−61.0%) NA	−411.322 (−38.9%) NA	−365.137 (−49.7%) NA	−312.685 (−35.3%) NA	−437.538 (−44.5%) NA	−437.538 (−37.9%) NA

The reduction in projected biomass of all size classes with depth generally reflects the exponential decline in supply of carbon to greater depths (Fig.2). However, there are generally greater reductions (as a percentage) in biomass of all size classes under both scenarios in deeper waters (Fig.2), particularly in the abyssal (>3000 m) and hadal zones (>6000 m). Under the RCP8.5 scenario, the relative reductions in biomass in deeper waters are particularly large (Fig.2), although it should be noted that the variability between the projections from separate ESMs is relatively high in these areas (Table S2).

In terms of regional responses, the Atlantic, Pacific, and Indian Oceans are projected to all experience major reductions in predicted overall export, POC flux and biomass in this century (Table 2). Regional trends are consistent between size classes and emissions scenarios, with larger changes under the more severe scenario (RCP8.5). The northeast Atlantic in particular is expected to have a broad area of reduced biomass, declining by as much as 38% in the Porcupine Abyssal Plain region. In the Atlantic, particular projected biomass reductions occur on the seafloor underlying the northern Gulf Stream and North Atlantic Drift currents as well as the Caribbean Sea (Fig.1 and Figure S1). Projected biomass increases occur in the Atlantic along the subtropical eastern seaboard of the United States and in the south Atlantic from the Cape Basin to southern Brazil. In the Pacific, the largest projected reductions occur in the Subtropical Gyres. There are also areas of reduced biomass following the North and South Equatorial Currents. The Pacific has localized increases in projected biomass off Chile and along the western seaboard of the United States. The Indian Ocean has projected reductions in benthic biomass throughout, with particular reductions in the Arabian Sea. Overall, the Polar Regions are the most consistent exceptions to the general trend of reducing benthic biomass with time, and both the Southern Ocean and the Arctic Ocean are projected to experience benthic biomass increases (Fig.1 and Figure S1), although variability in projections between individual ESMs was high (particularly in the Arctic; Table S2).

All of the important marine habitats and fishing areas investigated are projected to experience reductions in benthic metazoan biomass. Among the habitats investigated, areas with cold-water corals are projected to suffer the greatest declines and canyons the smallest declines (still >5%) in biomass (Tables 2 and S1). Out of a total of 8637 canyons identified by Harris & Whiteway (2011) 85% are projected to experience declines in benthic biomass in the next century. This includes important canyon systems in the North Atlantic, such as the Nazare Canyon (>14% decline in total biomass) and the North Pacific, such as the Monterey Canyon (>0.8% decline in total biomass). Some canyon systems, such as Barklay Canyon (>0.6% increase in total biomass) in the Pacific, are projected to increase in biomass. A total of 82% of the 33 452 individual seamounts recorded by Yesson *et al*. (2011) are projected to experience declines in biomass. Examples include Sedlo Seamount in the North Atlantic, the Graveyard Seamounts off New Zealand, and the seamounts of the northern Mid-Atlantic Ridge. A total of 94% of global cold-water coral reefs (Freiwald *et al.*, 2005) are projected to experience declines in benthic biomass. Over 93% of areas with the important reef-forming cold-water coral *Lophelia pertusa* and 97% with *Madrepora oculata* experience negative changes in total benthic biomass. Global deep-water fishing grounds (Watson *et al.*, 2004), as well as important regional fishing areas along the margin and seamounts of the North Atlantic (Devine *et al.*, 2006) and South Pacific (Clark & Dunn, 2012), are projected to experience large reductions in metazoan biomass. Reductions in biomass of fishing areas are projected to occur throughout the tropical oceans, although only small negative changes are projected for the Bay of Bengal and the eastern Indian Ocean. Limited change or even positive changes are projected for the few polar fishing areas, the Peru Basin, off north-west Africa, the northern Pacific, and western seaboard of the United States.

**Figure 3:**
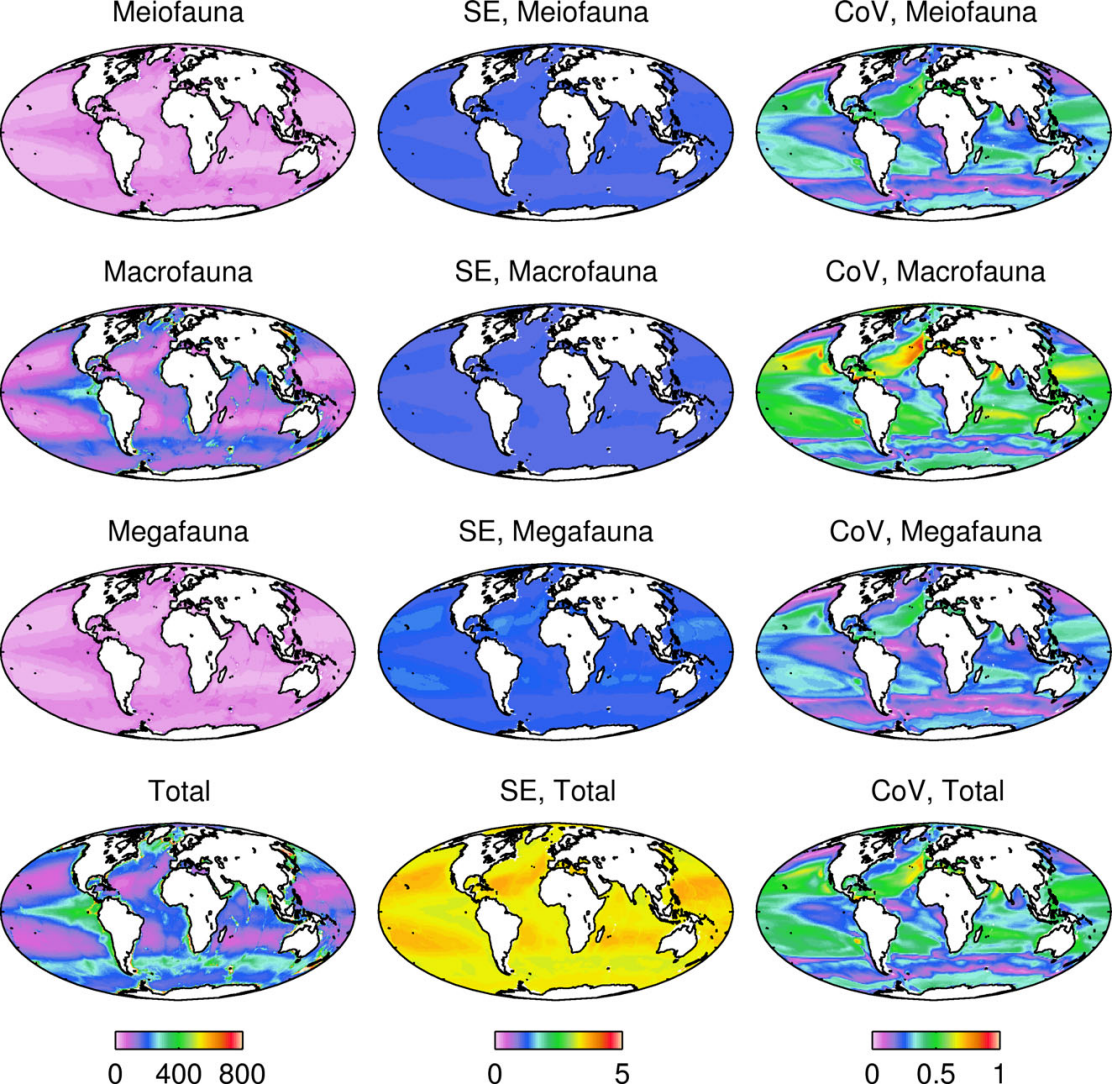
Biomass projections and error under RCP 8.5 for three metazoan size classes and total (mg C m^−2^) averaged for the period 2091–2100. Left column: biomass. Middle column: standard errors (SE) of regression relationship between biomass and flux. Right column: coefficient of variation (Cov) between eight model estimates of biomass.

**Figure 4:**
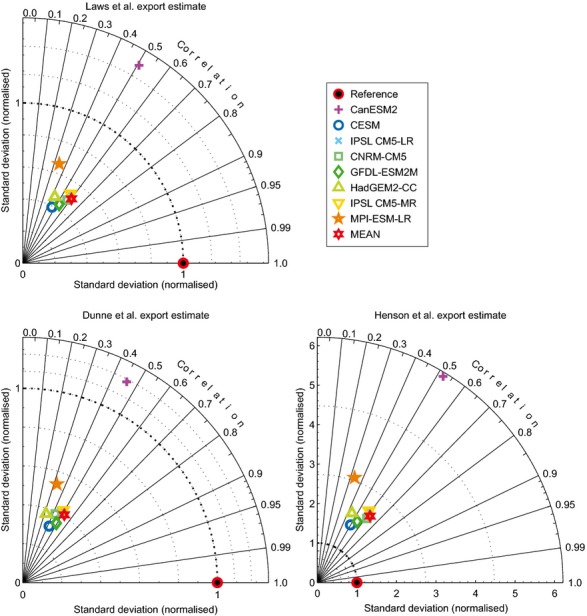
Comparison of modelled data and satellite-derived estimates of export production. Taylor (2001) diagrams display the correlation (circumference axis) and relative variability (radial axis) between the actual data (EP100obs) and model estimates (EP100). Comparisons are made between modelled export production (Figure S6) and Laws *et al*. (2000); Dunne *et al*. (2007) and Henson *et al*. (2012). The mean represents a simple mean of the eight other models.

The projections are reliant on the accuracy of the relationship between POC flux and the biomass size classes, as well as the overall quality of the models. The error associated with the regression equation was relatively low and not of sufficient magnitude to change the patterns observed (error ca. 1% of biomass of individual size classes; Table S2). The errors were higher in areas with low biomass (Figures S2–S4). The agreement among models (Figure S5; Table S2) and between models and independently derived data (Figs 3 and 4; Table S2) was generally good. For example, the global reduction in total biomass of 5.2 Mt C is associated with an error associated with the regression equation of 0.16 Mt C and a coefficient of variation between individual models of 0.40 Mt C. The maximum negative change projected for total biomass (−437.538 g C m^−2^) in the Atlantic has an error associated with the regression of 31.93 g C m^−2^ and a coefficient of variation between individual models of 0.80 g C m^−2^. It should also be noted that the use of a single *b* value for the Martin *et al*. (1987) algorithm, used to project flux to the seafloor and 500 m above the seafloor, may introduce additional error. This error may be exacerbated if climate-related changes in phytoplankton community structure and production (Buesseler & Boyd, 2009; Henson *et al.*, 2012) affects export flux attenuation or transfer efficiency.

When model output was compared with satellite-derived estimates of export (Figure S6), present day global totals of export in all eight models (4.47–10.63 Gt C yr^−1^; Table 1) were within the range of global totals of satellite-derived estimates (5.00–10.18 Gt C yr^−1^) (Laws *et al.*, 2000; Dunne *et al.*, 2007; Henson *et al.*, 2012). There was reasonable, if mixed, correlation between the modelled export flux and the satellite-derived estimates (Fig.4), although it should be noted that the satellite-based estimates are themselves derived from fitting an empirical relationship with a limited *in situ* dataset of export (Henson *et al.*, 2012). The multi-model mean always provided a closer correlation with independent satellite-based estimates than any individual model (Fig.4).

## Discussion

The benthic response to projected changes in the open ocean associated with climate change has not previously been quantified. The projections made here add significant detail to qualitative predictions (Smith *et al.*, 2008a), particularly in quantifying the magnitude and spatial patterns of changes. The projected changes in % biomass are greatest in the abyssal (>3000 m) and hadal zones (>6000 m) as a result of both higher relative changes in low-food conditions and the spatial co-occurrence of these areas with areas of change. These changes are likely to have a major impact as food supply to the benthos is already very low (Smith *et al.*, 2008a; Jamieson *et al.*, 2010) and limiting for benthic communities (Ruhl, 2008). These reductions will likely cause major changes in ecosystem structure, functioning and services across the largest habitat in the world (Smith *et al.*, 2008a).

The projected changes will result in a size-shift in global benthic biomass towards smaller organisms, particularly for the typically sediment-dwelling infaunal organisms (macro and meiofaunal). The decrease in size and biomass of infaunal organisms with reductions in flux (or increases in depth) has been observed for a long time (Thiel, 1975) and is likely simply because larger organisms require more energy than small organisms (Rex *et al.*, 2006). Evidence suggests that bacterial biomass is relatively constant across global surface sediments, including those underlying different productivity regimes (Rex *et al.*, 2006; Wei *et al.*, 2010). It is speculated, even in areas with low organic input, that bacterial assemblages may attach to settling particles and slowly accumulate to reach maximum density possible in a porous sedimentary matrix (Schmidt *et al.*, 1998). It is also likely that a proportion of this bacterial biomass is inactive, composed of dormant surface-derived species (Deming & Carpenter, 2008). Small changes in bacterial biomass in conjunction with decreases in metazoan biomasses could lead to a shift in abyssal benthic systems where bacteria are increasingly important in overall standing stock and energy flow. As well as overall reductions in biomass, increases in the proportion of small organisms may be expected, through allometry, to have several biological consequences, including increasing respiration rates for populations and reducing overall biomass production efficiency (Brown *et al.*, 2004; Smith *et al.*, 2008a; McClain *et al.*, 2012). Increases in small organisms may result in further additive impacts, such as reducing energy transfer to higher trophic levels (Brown *et al.*, 2004). These processes may affect the rate of change of benthic ecosystems in future scenarios, particularly if they are associated with concurrent changes in ambient temperature (McClain *et al.*, 2012). Pelagic ecosystems in surface oceans are similarly projected to experience major size-reductions as well as overall biomass reductions with future climate change (Cheung *et al.*, 2013). Benthic megafaunal organisms, here representing large epifauna and demersal fish (ranging from grams to tens of kilograms wet weight biomass), are not projected to experience as large a declines as for macrofauna (although similar to meiofauna). Although few mechanistic insights are provided by the empirical relationships used, the differences in feeding mode of epifaunal and demersal megafauna compared to infaunal organisms may explain the smaller reductions in biomass into the future. For example the deposit feeding megafauna, such as holothurians, are able to rapidly remove newly deposited POC (Lauerman *et al.*, 1997; Miller *et al.*, 2000) and scavenging megafauna, such as demersal fishes, can quickly access larger organic food-falls (Collins *et al.*, 2005; Rex *et al.*, 2006), both potentially ameliorating the impact of reducing POC flux to this group.

Differential changes within (and potentially between) ocean basins will likely lead to major changes in the spatial distribution of benthic species, with subsequent impacts on biodiversity. The best example is the North Atlantic, an area of major projected change, with generally more positive changes in benthic biomass on the western side and major negative changes to the east. Of the few deep-water species investigated, many have high connectivity of populations, even when separated by large features such as mid-ocean ridges (Priede *et al.*, 2013), so range shifts seem likely to occur in response to changes in flux. Future changes, particularly the positive changes in biomass projected in the polar regions (Tables 2 and S1), may facilitate colonization by invasive species (Thatje, 2005), particularly when associated with synergistic changes in other parameters, including temperature, dissolved oxygen and pH (Mora *et al.*, 2013). Elevated supply of organic matter to the seafloor may also decrease biodiversity, as fast-responding colonists dominate communities (Levin *et al.*, 2001).

Potential biodiversity hotspots are projected to experience biomass decreases (Tables 2 and S1), which could have major consequences for the species they harbour (Smith *et al.*, 2008a). More than 80% of the high-habitat heterogeneity systems known around the world, including canyons, seamounts, and cold-water coral reefs, are projected to experience negative changes in biomass. These habitats are very important for deep-sea ecosystem functioning and provide many useful services to humans. Our projections are also likely underestimates in these geologically complex areas as they do not take into account subsurface processes, such as lateral transport of organic material, which are often important (Wei *et al.*, 2010, 2012). As an example, canyons channel and concentrate detrital matter, enhancing local benthic biomass and fishery production (De Leo *et al.*, 2010), and will therefore likely experience more extreme local changes in biomass than the coarse-resolution projections made here. A large proportion of the world's seamounts are projected to experience reductions in biomass; these are areas that support major fisheries and are already highly exploited and susceptible to human activities (Clark *et al.*, 2010). Cold-water corals are ecosystem engineers, recognized as providing important habitats, and are conservation priorities across the world (Davies & Guinotte, 2011). Cold-water coral reefs tend to occur in areas with high local food supply (Davies & Guinotte, 2011) and it is likely that they will be easily affected by negative changes in flux. Although the reef structure can persist for some time, still providing habitat (Wheeler *et al.*, 2005), the long-term effects of biomass reductions in the >90% of coral reef habitats may be severe, especially with the additional negative impacts of projected ocean acidification (Gehlen *et al*., submitted).

Major declines in biomass are predicted in areas currently supporting high deep-water fishing activity, as a result of global-scale changes in flux to the seafloor. The methods used here, however, do not include variable fishing pressure (Devine *et al.*, 2006), which would likely alter our projections. Although both climatic change and fishing may have similar consequences for fisheries, reducing biomass and leading to size-shifts toward smaller organisms (Cheung *et al.*, 2010), it is possible that reductions in the biomass of higher trophic levels from fishing may mitigate climate change-induced impacts by reducing top-down control on the organisms at lower trophic levels.

The results also provide information useful in planning future monitoring. The existing open ocean benthic time-series observations show major variations in community composition of abyssal systems across the size range of benthic fauna investigated, related to changes in quantity and quality of POC flux (Billett *et al.*, 2001; Smith *et al.*, 2008b). The Porcupine Abyssal Plain time-series station (Billett *et al.*, 2001) in the north Atlantic lies within an area of predicted major change [projections suggest a total metazoan biomass reduction of 78.48 mg C m^−2^ or −38.5% under RCP8.5 (40.47 mg C m^−2^ or −19.5% under RCP4.5)]. In contrast, the Pacific Station M time-series (Smith *et al.*, 2008b) is projected to experience considerably smaller changes (total metazoan biomass increase of 1.14 mg C m^−2^, +0.4% under RCP4.5; and decrease of 4.91 mg C m^−2^, −1.8% under RCP8.5). Some polar time-series stations, such as the Arctic HAUSGARTEN [total metazoan biomass decrease of 11.13 mg C m^−2^ or −4.0% under RCP8.5 (4.99 mg C m^−2^ or −1.8% under RCP4.5)] (Bergmann *et al.*, 2011), are projected to loose biomass. Others, such as the Antarctic FOODBANCS [total metazoan biomass increase of 47.19 mg C m^−2^ or +5.1% RCP8.5 (18.35 mg C m^−2^ or +1.9% RCP4.5)] (Smith *et al.*, 2012) sites, are projected to experience positive changes. As such, these major time-series stations may represent good model systems to investigate the variability among regional impacts of projected changes.

The World Ocean seafloor fauna are expected to change substantially with climate change, although these changes may have different implications for different regions. The changes in food supply, mediated through changes in surface ocean producers (Boyce *et al.*, 2010) and alterations to the physical properties of the future water column, may have profound effects on deep-water ecosystems (Smith *et al.*, 2008a). Reductions in food supply may lead to potential responses at the population and individual level, such as reductions in adult body size (McClain *et al.*, 2005), longevity (Gillooly *et al.*, 2002), and reproductive success (Young & Eckelbarger, 1994). The species energy hypothesis suggests that reductions in potential energy and smaller population sizes may lead to reductions in biodiversity (Allen *et al.*, 2007). Reductions in POC flux over broad areas may even lead to regional or global extinctions as population standing stocks decrease beyond reproductively viable levels (Rex *et al.*, 2006).

Future projections of changes near the seabed of the open ocean include manifold impacts, including broad increases in temperature, decreased oxygen, lowering pH (Mora *et al.*, 2013) and introduction of chemical pollutants as well as the changes in benthic food supply that are the focus of this paper. The additive effects of changes in all these parameters may extend the impacts on biomass beyond the projections made here and should be the focus of future work. Nonetheless, owing to the strong dependence of benthic ecosystems on surface productivity and export, and their probable change into the future, changes in POC fluxes are likely to have the primary impact on these systems.

## Supporting Information

Table S1 Changes in POC flux and biomass between 2006–2015 and 2091–2100 under moderate scenario (RCP4.5). **Table S2.** Error associated with projections of POC flux and biomass between 2006–2015 and 2091–2100 under severe scenario (RCP8.5). **Figure S1.** Changes in benthic biomass between 2006–2015 and 2091–2100 under moderate scenario (RCP4.5). **Figure S2.** Biomass projections and error under RCP4.5 for three metazoan size classes and total averaged for the period 2006–2015. **Figure S3.** Biomass projections and error under RCP4.5 for three metazoan size classes and total averaged for the period 2091–2100. **Figure S4.** Biomass projections and error under RCP8.5 for three metazoan size classes and total averaged for the period 2006–2015. **Figure S5.** Separate model projections of export (from 100 m) for the period 2006–2016 (RCP4.5 and 8.5 are very similar close to present times). **Figure S6.** Comparison of multi-model mean export (from 100 m) averaged for the period 2006–2015 with satellite-derived independent export estimates from Laws *et al*., 2000; Dunne *et al*., 2007 and Henson *et al*., 2012.
