# Supplementary material for: Global reductions in seafloor biomass in response to climate change
Source: Glob Chang Biol. 2013 Dec 31;20(6):1861–72. doi: 10.1111/gcb.12480 (PMC4261893; doi:10.1111/gcb.12480)
Supplement: Table S1 — Changes in POC flux and biomass between 2006–2015 and 2091–2100 under moderate scenario (RCP4.5). Table S2. Error associated with projections of POC flux and biomass between 2006–2015 and 2091–2100 under severe scenario (RCP8.5). Figure S1. Changes in benthic biomass between 2006–2015 and 2091–2100 under moderate scenario (RCP4.5). Figure S2. Biomass projections and error under RCP4.5 for three metazoan size classes and total averaged for the period 2006–2015. Figure S3. Biomass projections and error under RCP4.5 for three metazoan size classes and total averaged for the period 2091–2100. Figure S4. Biomass projections and error under RCP8.5 for three metazoan size classes and total averaged for the period 2006–2015. Figure S5. Separate model projections of export (from 100 m) for the period 2006–2016 (RCP4.5 and 8.5 are very similar close to present times). Figure S6. Comparison of multi-model mean export (from 100 m) averaged for the period 2006–2015 with satellite-derived independent export estimates from Laws et al., 2000; Dunne et al., 2007 and Henson et al., 2012. [file gcb0020-1861-SD1.doc]

**Supplementary Table**

Supplementary Table 1: Changes in POC flux and biomass between 2006-2015 and 2091-2100 under moderate scenario (RCP4.5). Changes in specific regions and depth bands for POC flux to seafloor (Mt C y^-1^) and biomass (MtC). Values presented are absolute change between present and future projections. The greatest positive and negative changes are local values (per 1° cell) in units of mol C m^-2^ y^-1^ for POC flux and g C m^-2^ for biomass. Percentage changes are presented in parentheses. For local areas of greatest positive and negative change regional location codes are given: NA: North Atlantic, Ar: Arctic.

| Area | POC flux to seafloor | Meiofauna | Macrofauna | Megafauna | Metazoan Total | Total (with bacteria constant) |
| --- | --- | --- | --- | --- | --- | --- |
| Globe | -22.440  (-4.44%) | -0.308  (-2.34%) | -1.502  (-3.34%) | -0.277  (-2.05%) | -2.087  (-2.91%) | -2.087  (-2.08%) |
| Atlantic | -11.408  (-6.86%) | -0.110  (-3.33%) | -0.552  (-4.79%) | -0.098  (-2.92%) | -0.760  (-4.18%) | -0.760  (-3.07%) |
| Pacific | -14.886  (-1.84%) | -0.303  (-1.21%) | -1.434  (-1.70%) | -0.276  (-1.06%) | -2.013  (-1.49%) | -2.013  (-1.05%) |
| Indian | -9.603  (-1.41%) | -0.189  (-0.81%) | -0.890  (-1.12%) | -0.171  (-0.71%) | -1.250  (-0.98%) | -1.250  (-0.68%) |
| Arctic | -1.552  (-4.95%) | 0.0004  (0.185%) | -0.018  (-2.11%) | 0.001  (0.580%) | -0.017  (-1.20%) | -0.017  (-0.97%) |
| Southern | 1.128  (3.76%) | 0.016  (2.14%) | 0.080  (3.09%) | 0.014  (1.86%) | 0.110  (2.69%) | 0.110  (1.92%) |
| Bathyal | -6.238  (-4.42%) | -0.035  (-2.17%) | -0.244  (-3.16%) | -0.029  (-1.88%) | -0.308  (-2.83%) | -0.308  (-2.26%) |
| Abyssal | -12.672  (-4.83%) | -0.270  (-2.36%) | -1.251  (-3.38%) | -0.246  (-2.07%) | -1.768  (-2.93%) | -1.768  (-2.05%) |
| Hadal | -0.052  (-3.64%) | -0.002  (-2.48%) | -0.007  (-3.13%) | -0.002  (-2.24%) | -0.011  (-2.80%) | -0.011  (-1.84%) |
| Area with fishing | -9.849  (-6.11%) | -0.038  (-2.89%) | -0.230  (-4.05%) | -0.032  (-2.56%) | -0.300  (-3.64%) | -0.300  (-2.73%) |
| Area with seamounts | -6.430  (-5.44%) | -0.141  (-2.79%) | -0.647  (-3.93%) | -0.129  (-2.45%) | -0.916  (-3.43%) | -0.916  (-2.40%) |
| Area with canyons | -4.071  (-4.85%) | -0.044  (-2.54%) | -0.256  (-3.59%) | -0.037  (-2.23%) | -0.337  (-3.21%) | -0.337  (-2.34%) |
| Area with cold- water corals | -2.735  (-10.7%) | -0.012  (-4.06%) | -0.066  (-5.44%) | -0.011  (-3.62%) | -0.089  (-4.91%) | -0.089  (-3.78%) |
| Greatest positive change | 117254  (42.61%) Ar | 82.800  (18.85%) Ar | 88.973  (27.10%) Ar | 59.661  (16.45%) Ar | 1032.195  (22.70%) Ar | 1032.195  (22.70%) Ar |
| Greatest negative change | -204314  (-49.6%) NA | -255.837  (-31.1%) NA | -233.188  (-40.4%) NA | -192.942  (-27.9%) NA | -278.066  (-36.2%) NA | -278.066  (-28.3%) NA |

**Supplementary Table 2: Error associated with projections of POC flux and biomass between 2006-2015 and 2091-2100 under severe scenario (RCP8.5).** Errors associated with projections of changes in specific regions and depth bands for POC flux to seafloor (Mt C y^-1^) and biomass (MtC). Values presented are error associated with regression equation (Reg. Er.) and coefficient of variation (CoV) between models for absolute changes between present and future projections. This table provides error estimates associated with the absolute values in Table 1 in the main paper. Totals were the same with and without the constant bacteria.

|  | POC flux to seafloor | Meiofauna | | Macrofauna | | Megafauna | | Total | |
| --- | --- | --- | --- | --- | --- | --- | --- | --- | --- |
|  | CoV | Reg. Er. | CoV | Reg. Er. | CoV | Reg. Er. | CoV | Reg. Er. | CoV |
| Globe | 0.577 | 0.048 | 0.415 | 0.020 | 0.363 | 0.136 | 0.439 | 0.163 | 0.374 |
| Atlantic | 0.528 | 0.017 | 0.373 | 0.002 | 0.336 | 0.048 | 0.391 | 0.063 | 0.343 |
| Pacific | 0.491 | 0.043 | 0.323 | 0.026 | 0.280 | 0.127 | 0.351 | 0.143 | 0.284 |
| Indian | 0.591 | 0.030 | 0.636 | 0.023 | 0.605 | 0.089 | 0.647 | 0.096 | 0.614 |
| Arctic | 2.030 | 0.001 | 15.263 | 0.003 | 3.548 | 0.002 | 5.887 | 0.006 | 5.531 |
| Southern | 0.514 | 0.002 | 0.418 | <0.001 | 0.399 | 0.006 | 0.428 | 0.008 | 0.402 |
| Bathyal | 0.714 | 0.011 | 0.509 | 0.019 | 0.495 | 0.024 | 0.517 | 0.054 | 0.497 |
| Abyssal | 0.397 | 0.036 | 0.413 | 0.038 | 0.356 | 0.112 | 0.439 | 0.110 | 0.368 |
| Hadal | 0.603 | <0.001 | 0.635 | 0.001 | 0.610 | 0.001 | 0.644 | <0.001 | 0.618 |
| Area with fishing | 0.877 | 0.002 | 0.486 | 0.004 | 0.453 | 0.004 | 0.499 | 0.010 | 0.458 |
| Area with seamounts | 0.350 | 0.018 | 0.425 | 0.019 | 0.358 | 0.057 | 0.450 | 0.057 | 0.375 |
| Area with canyons | 0.476 | 0.010 | 0.482 | 0.009 | 0.458 | 0.023 | 0.492 | 0.043 | 0.463 |
| Area with cold- water corals | 0.629 | 0.002 | 0.326 | 0.001 | 0.342 | 0.006 | 0.329 | 0.009 | 0.334 |

**Supplementary Figures**


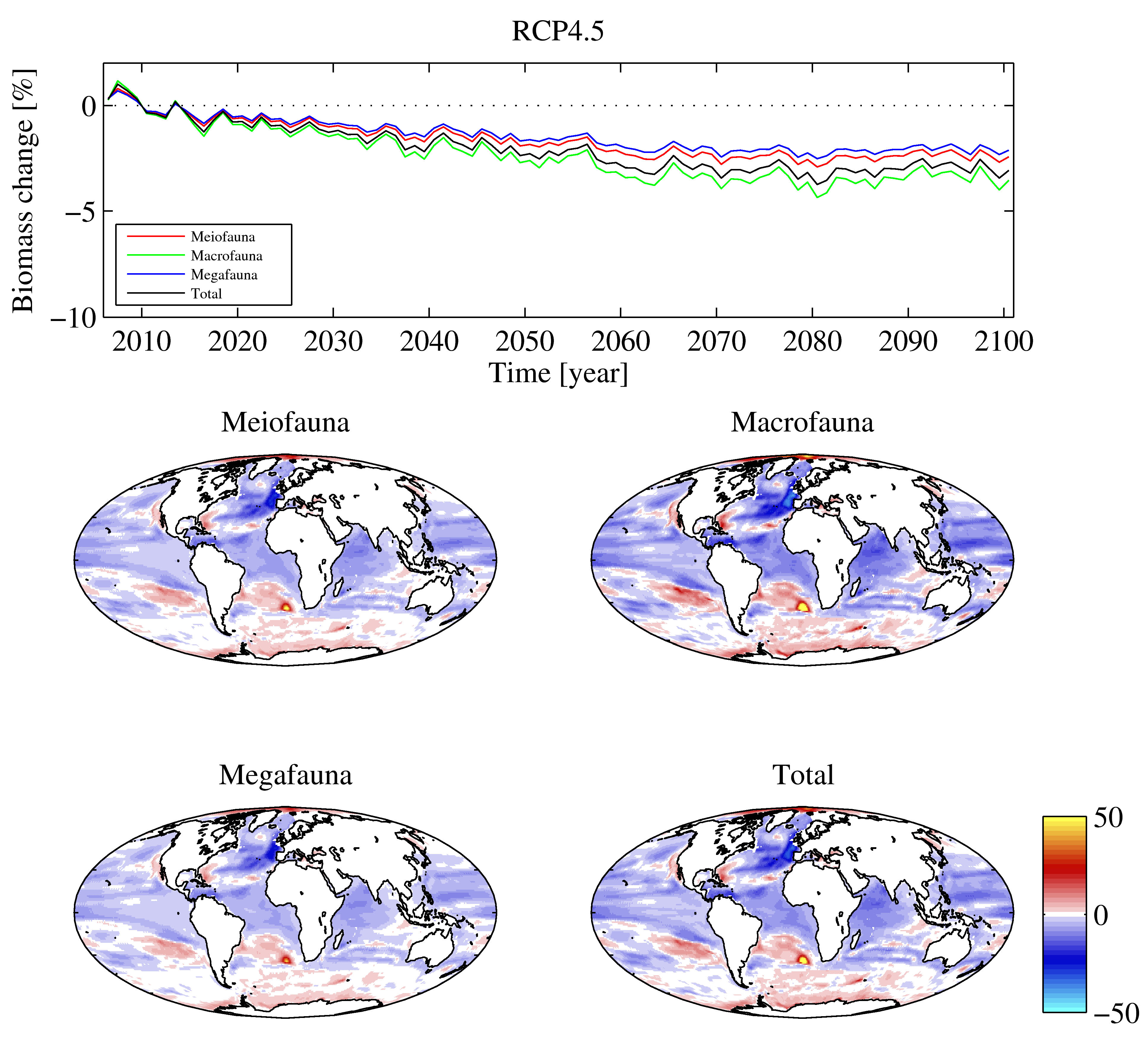


**Supplementary Figure 1: Changes in benthic biomass between 2006-2015 and 2091-2100 under moderate scenario (RCP4.5).** Panel 1 shows projected changes in biomass of metazoan size-categories of benthos across the modelled time series (as annual means). Panels 2-5 show maps of percentage changes in multi-model mean benthic biomass on seafloor (mg C m^-2^). Benthic biomasses presented as totals (metazoans only) and split into three size-classes.


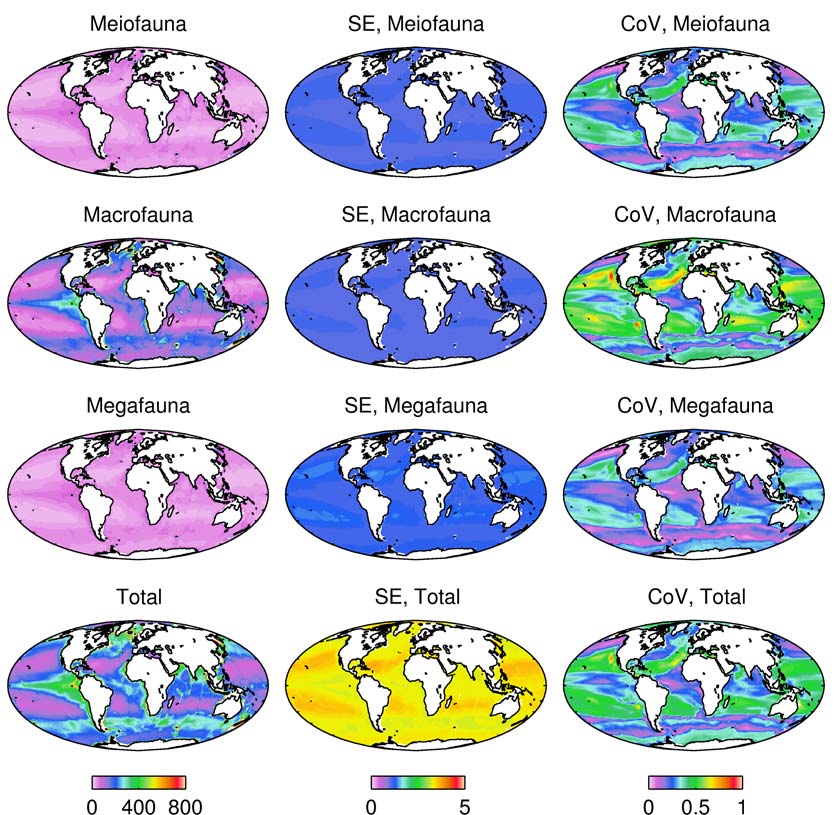


**Supplementary Figure 2: Biomass projections and error under RCP 4.5 for three metazoan size classes and total averaged for the period 2006-2015.** Left column: Biomass. Middle column: Standard errors (SE) of regression relationship between biomass and flux. Right column: Coefficient of Variation (Cov) between eight model estimates of biomass.


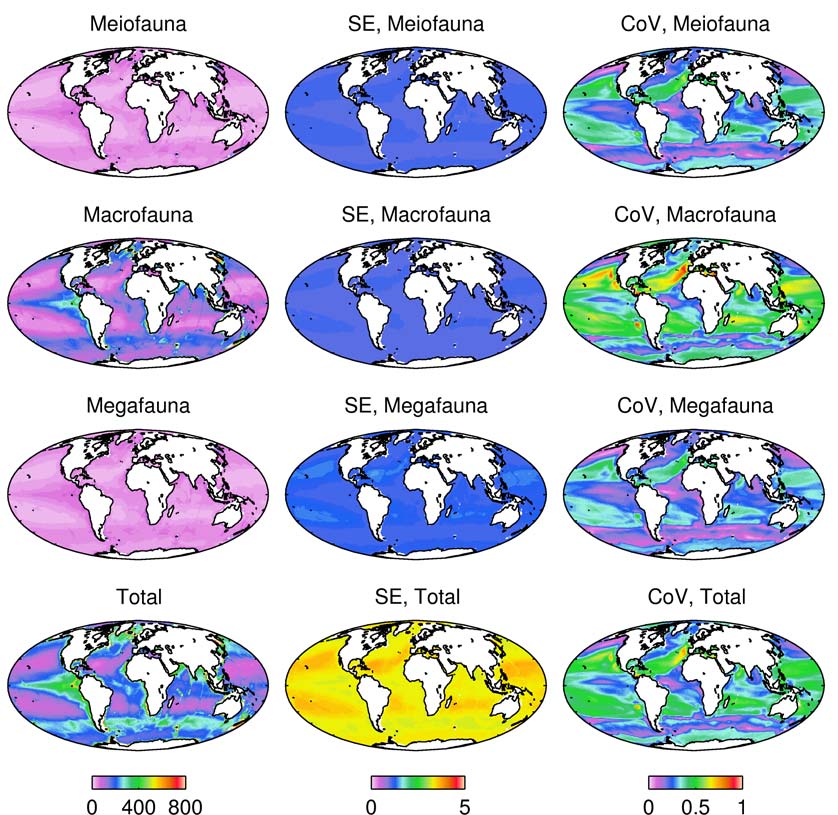


**Supplementary Figure 3: Biomass projections and error under RCP 4.5 for three metazoan size classes and total averaged for the period 2091-2100.** Left column: Biomass. Middle column: Standard errors (SE) of regression relationship between biomass and flux. Right column: Coefficient of Variation (Cov) between eight model estimates of biomass.


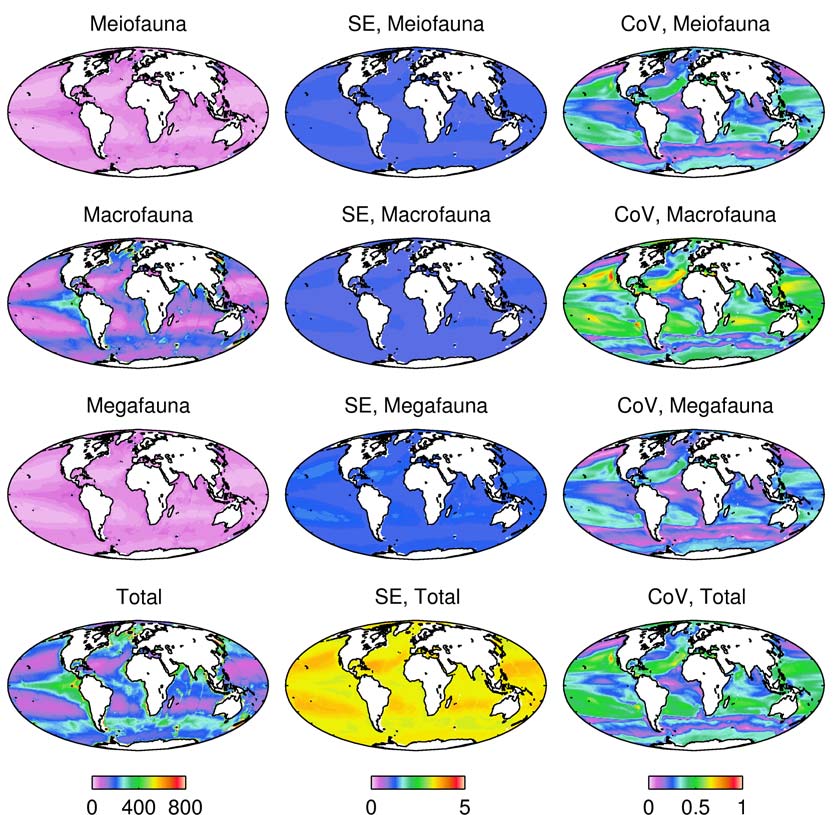


**Supplementary Figure 4: Biomass projections and error under RCP 8.5 for three metazoan size classes and total averaged for the period 2006-2015.** Left column: Biomass. Middle column: Standard errors (SE) of regression relationship between biomass and flux. Right column: Coefficient of Variation (Cov) between eight model estimates of biomass.


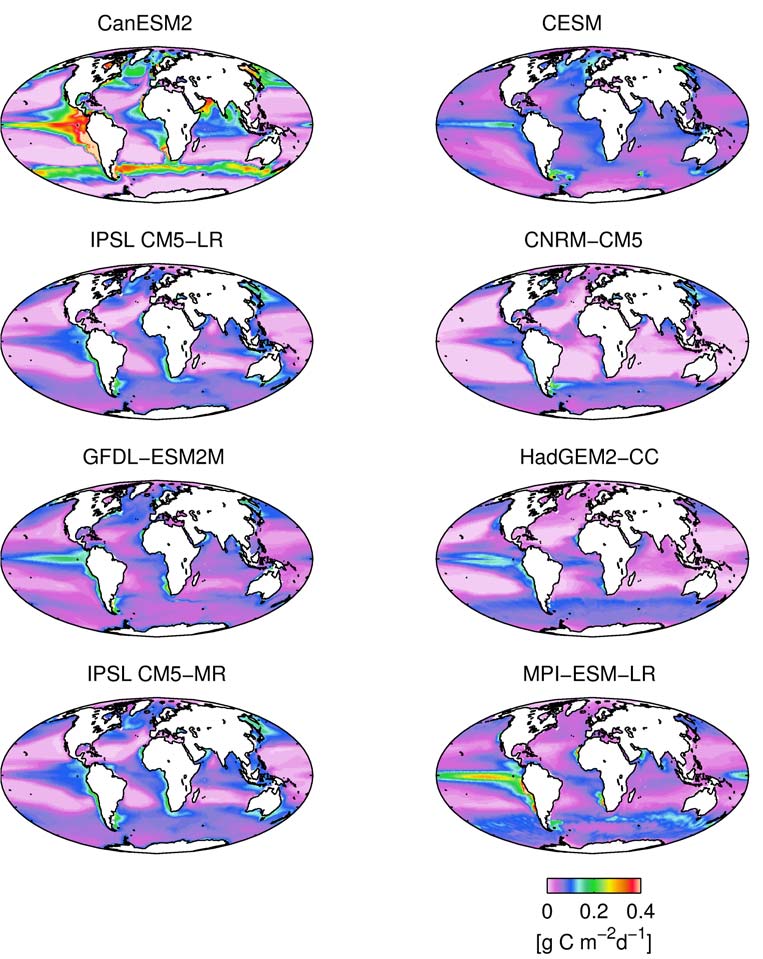


**Supplementary Figure 5: Separate model projections of export (from 100m) for the period 2006-2016 (RCP4.5 and 8.5 are very similar close to present times).**


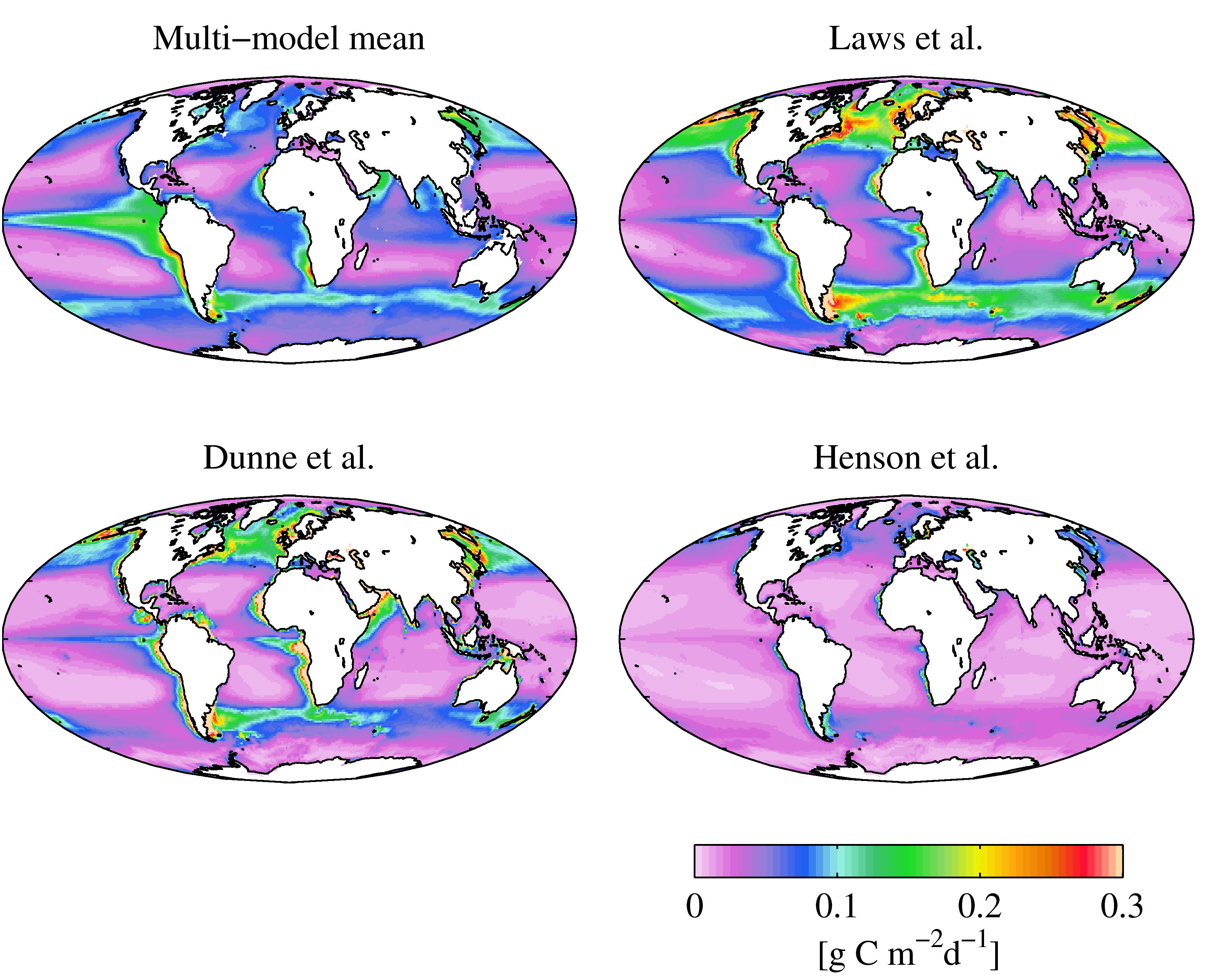


**Supplementary Figure 6: Comparison of multi-model mean export (from 100m) averaged for the period 2006-2015 with satellite-derived independent export estimates from Laws et al., 2000, Dunne et al., 2007 and Henson et al., 2012.**
